# Supplementary figures and images for: Methylomic analysis identifies C11orf87 as a novel epigenetic biomarker for GI cancers
Source: PLoS One. 2021 Apr 22;16(4):e0250499. doi: 10.1371/journal.pone.0250499 (PMC8062079; doi:10.1371/journal.pone.0250499)

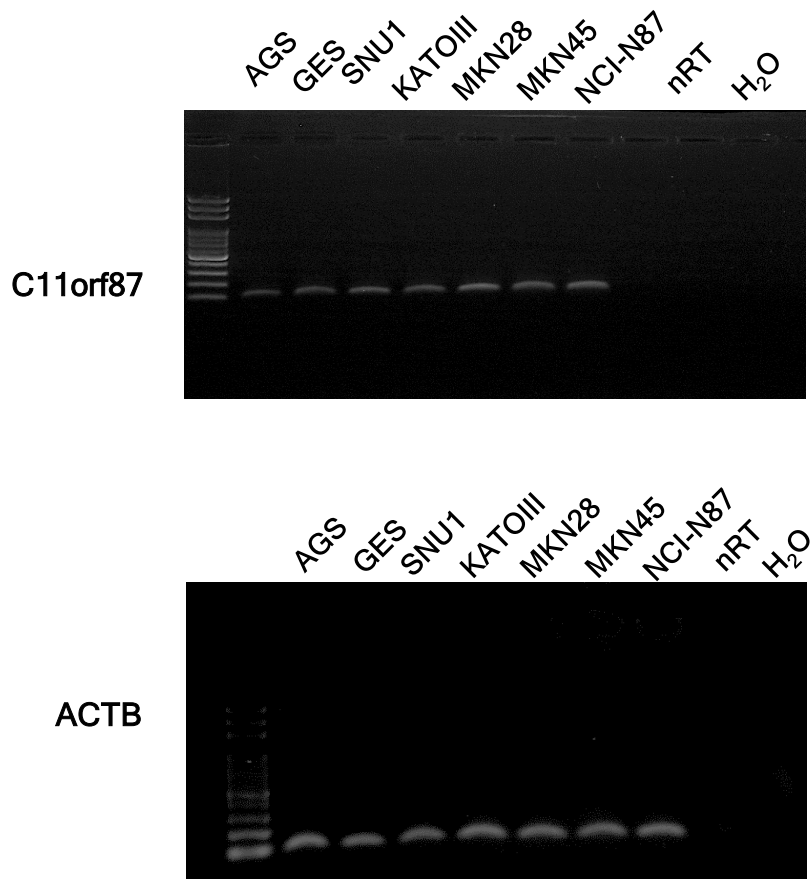

Supplement: S1 Fig — (PDF) [file pone.0250499.s001.pdf]
