# Supplementary material for: Methylomic analysis identifies C11orf87 as a novel epigenetic biomarker for GI cancers
Source: PLoS One. 2021 Apr 22;16(4):e0250499. doi: 10.1371/journal.pone.0250499 (PMC8062079; doi:10.1371/journal.pone.0250499)
Supplement: Attachment — Submitted filename: Tran et al_C11orf87_response_rev.pdf [file pone.0250499.s002.pdf]

Point-to-point responses

<u>Comments from Reviewer #1:</u>

We thank the reviewer for the insightful review and interest in our manuscript. We have now carefully read each comment, and incorporated the recommendations and suggestions in the following manner:

1. The authors postulated the relationship of STAT3 activation with epigenetically silenced C11orf87 in GI cancers. However, some key experiments should be performed to identify this conclusion, such as the effect of C11orf87 methylation and expression in gastric cell lines with STAT3 reactivation.

Response: We thank the review for this important question. We now performed additional experiments to investigate the relationship between C11orf87 methylation and expression in a panel of gastric cancer cell lines. The results showed that those cell lines exhibited various *C11orf87* expression (Fig. 2A). Unexpectedly, further bisulfite pyrosequencing also showed various *C11orf87* methylation without correlation with its expression (Fig. 2B). These results suggested that *C11orf87* expression is independent of its methylation.

These new results can be found in Figure 2A, B and Page 7 of the result section.

2. The C11orf87 expression and methylation levels in GI cell lines with different STAT3 status should be examined.

Response: Again, we thank the reviewer for this important question. We now performed additional experiments to examine the effect of STAT3 on C11orf87 expression. We ectopically expressed a constitutive active STAT3 mutant (STAT3c) in MKN28, a STAT3 inactive cell line. However, ectopic expression of STAT3c promoted *C11orf87* expression in MKN28 cells (Fig. 2C), without affecting its methylation (Fig. 2D). In this regard, we hypothesized that STAT3 may contribute to methylation maintenances rather than de novo methylation. Indeed, treatment of STAT3 inhibitor, JSI-124, resulted in a downregulation of C11orf87 expression (Fig. 2E). Taken together, these results suggested that the C11orf87 methylation may be a passenger effect under the STAT3-mediated C11orf87 expression.

These new results can be found in Figure 2C-E and Page 7 of the Result sections.

3. What is the expression pattern of C11orf87 in GI tumor tissues? Is there any correlation with its methylation level?

Response: We thank the reviewer for this question. Unfortunately, we don’t have any RNA of the in-house tumor tissue samples to perform such experiments. We therefore performed additional experiments to investigate the relationship between *C11orf87* methylation and expression in a panel of gastric cancer cell lines. The results showed that those cell lines exhibited various *C11orf87* expression (Fig. 2A). Unexpectedly, further bisulfite pyrosequencing also showed various *C11orf87* methylation without correlation with their expression (Fig. 2B). These results

suggested that *C11orf87* expression is independent of its methylation. As the methylation wasn't crucial for controlling C11orf87 expression, we then investigated whether the methylation of *C11orf87* could serve as a biomarker for gastric cancer. Interestingly, we found that *C11orf87* methylation can be an epigenetic biomarker for GI cancers.

4. As a novel biomarker of GI cancers, what are the biological functions of C11orf87 in GI cells?

Response: We thank the reviewer for this important question. C11orf87, known as neuronal integral membrane protein 1, was found to be predominantly expressed in the brain tissue. However, the involvement of C11orf87 in human cancer has not been characterized. A recent study in head and neck cancer found that p53 mutated tumors could promote differentiation of nerve fibers, which then promoted tumor growth in this tumor microenvironment [1]. As the expression of *C11orf87* was controlled by STAT3, we, therefore, postulate that aberrant STAT3 activation may involve in promoting differentiation of nerve fibers via upregulation of *C11orf87*. Although p53 mutation is frequent in gastric cancer [2], how neuronal-related gene control gastric cancer progression still requires further investigation.

These statements have been added in Page 11 of the Discussion section.

Comments from Reviewer #2:

We thank the reviewer for the insightful review and interest in our manuscript. We have now carefully read each comment, and incorporated the recommendations and suggestions in the following manner:

1.The detection results of different methylation sequencing methods are very different. Are the methylation detection methods used in the TCGA and GSE103186 database the same? Is the combined analysis reasonable?

Response: We thank the reviewer for this important question. We apologize that we didn't state clearly the methodology of these two dataset. Methylation analysis of TCGA and GSE103186 dataset are indeed from the same microarray platform (Infinium HumanMethylation450 Beadchip). Therefore, we combined these two dataset for the analyses. We have added a statement to clarify they are indeed from the same microarray platform (Page 8 of the Result section).

2.Because the authors screened the target genes of STAT3 through DNA methylation microarray, please analyze and verify the relationship between STAT3 and C11orf87.

Response: Again, we thank the reviewer for this important question. We now performed additional experiments to examine the effect of STAT3 on *C11orf87* expression. We ectopically expressed a constitutive active STAT3 mutant (STAT3c) in MKN28, a STAT3 inactive cell line. However, ectopic expression of STAT3c promoted *C11orf87* expression in MKN28 cells (Fig. 2C), without affecting its methylation (Fig. 2D). In this regard, we hypothesized that STAT3 may contribute to methylation maintenances rather than de novo methylation. Indeed, treatment of STAT3 inhibitor, JSI-124, resulted in a downregulation of C11orf87 expression (Fig. 2E). Taken together, these results suggested that the C11orf87 methylation may be a passenger effect under the STAT3-mediated C11orf87 expression.

3.What is the expression of C11orf87 in TGCA database and in-house samples? The correlation analysis between its methylation and expression? And, the correlation analysis between its expression and prognosis?

Response: We thank the reviewer for this question. Unfortunately, we don't have any RNA of our in-house tumor tissues to perform such experiments. As the methylation wasn't crucial for controlling C11orf87 expression, we then investigated whether the methylation of C11orf87 could serve as a biomarker for gastric cancer. Interestingly, we found that C11orf87 methylation can be an epigenetic biomarker for GI cancers.

These new results can be found in Figure 2 and Page 7 of the result section.

4.In a variety of gastrointestinal tumors, including gastric cancer, the methylation level of C11orf87 in cancer tissues is higher than that in adjacent tissues and/or normal tissues. Therefore, the author proposes that C11orf87 may be used as a biomarker for gastric cancer. However, gastric cancer patients with high C11orf87 methylation have a better prognosis, which seems to be contrary to previous results. The authors think that it may be caused by the role of STAT3 in gastric cancer, but lack of relevant experimental results.

Response: We thank the reviewer for this important question. As mentioned in point #2 above, we found that ectopic expression of STAT3c promoted C11orf87 expression in MKN28 cells, without affecting its methylation. While treatment of STAT3 inhibitor, JSI-124, resulted in a downregulation of *C11orf87* expression. Taken together, these results suggested that the *C11orf87* methylation may be a passenger effect under the STAT3-mediated C11orf87 expression.

Recently, a study demonstrated that SIRT1, a histone deacetylase that participated in STAT3 deacetylation, was found to be upregulated in advanced gastric cancer [3]. The authors suggested that SIRT1 upregulation may compensate for the damaging effect induced by constitutive activation of STAT3 in gastric cancer. In this regard, SIRT1 may disrupt the interaction between STAT3 and DNMT1 by deacetylation on Lys685, which further limited methylation maintenances. Herein, we postulated that hypermethylation of C11orf87 may serve as a “vestigial marker” for constitutive activation of STAT3 in gastric cancer. This hypothesis may further explain our clinical observation that *C11orf87* hypermethylation was related to better survival.

We have added those statement in page 10 of the Discussion section.

References

1. Amit, M., et al., *Loss of p53 drives neuron reprogramming in head and neck cancer.* Nature, 2020. **578**(7795): p. 449-454.
2. Rhyu, M.G., et al., *Allelic deletions of MCC/APC and p53 are frequent late events in human gastric carcinogenesis.* Gastroenterology, 1994. **106**(6): p. 1584-8.
3. Zhang, S., et al., *SIRT1 inhibits gastric cancer proliferation and metastasis via STAT3/MMP-13 signaling.* J Cell Physiol, 2019. **234**(9): p. 15395-15406.
