# Supplementary material for: Methylomic analysis identifies C11orf87 as a novel epigenetic biomarker for GI cancers
Source: PLoS One. 2021 Apr 22;16(4):e0250499. doi: 10.1371/journal.pone.0250499 (PMC8062079; doi:10.1371/journal.pone.0250499)
Supplement: Attachment — Submitted filename: response_rev.pdf [file pone.0250499.s003.pdf]

Point-to-point responses

<u>Comments from Reviewer #1:</u>

We thank the reviewer for the insightful review and interest in our manuscript. We have now carefully read each comment, and incorporated the recommendations and suggestions in the following manner:

1. The labeling is misplaced in Figure 2A.

Response: We apologize for this mistake, we have now aligned the labeling in the correct place.

2. The authors identified a potential STAT3 target, C11orf87, through using DNA methylation microarray, which is hypomethylated in gastric cancer patients with lower STAT3 activation and AGS cells with STAT3 inactivation. The authors thus inferred a possible correlation between C11orf87 methylation/expression and STAT3 activation. However, after ectopic expression of STAT3c, no methylation changes of C11orf87 was found in MKN28 with STAT3 inactivation (Figure 2D), and also no bars were shown. The authors should repeat the experiment and do statistical analysis (Figure 2B, 2D). The methylation analysis of C11orf87 in cells with the treatment of STAT3 inhibitor JSI-124 should also be examined.

Response: We thank the reviewer for this important question. We have repeated the bisulfite pyrosequencing in Fig 2B and D, and the results are very consistent, showing low SD. We have replaced Fig 2B and D with error bars. Regarding AGS cells treated with STAT3 inhibitor (JSI-124), we have now performed bisulfite pyrosequencing to examine the changes of C11orf87 methylation. Surprisingly, treatment of JSI-124 did not affect C11orf87 methylation in AGS cells (Fig. 2F). This results suggested that long term STAT3 depletion may be required to disrupt C11orf87 methylation, as previously observed in NR4A3, a STAT3-downregulated target [1].

This new result can be found in Fig 2F and Page 7 of the Result section.

References

1. Yeh CM, Chang LY, Lin SH, Chou JL, Hsieh HY, et al. (2016) Epigenetic silencing of the NR4A3 tumor suppressor, by aberrant JAK/STAT signaling, predicts prognosis in gastric cancer. Sci Rep 6: 31690.

Comments from Reviewer #2:

We thank the reviewer for the insightful review and interest in our manuscript. We have now carefully read each comment, and incorporated the recommendations and suggestions in the following manner:

1. Fig2a. there are 9 samples, but only 8 bands for C11orf87. 7 binds for Actin.

Response: We apologize for this mistake, we have now aligned the labeling in the correct place.

2.Fig2c,2d. please detect expression of C11orf87 by qRT-PCR.

Response: Expression of C11orf87 in Fig 2C and E is now replaced by qRT-PCR. Thank you for this comment.
